# Biosynthesis of Silver Nanoparticles by Marine Actinobacterium *Nocardiopsis dassonvillei* and Exploring Their Therapeutic Potentials

**DOI:** 10.3389/fmicb.2021.705673

**Published:** 2022-02-03

**Authors:** Maha A. Khalil, Abd El-Raheem R. El-Shanshoury, Maha A. Alghamdi, Fatin A. Alsalmi, Samia F. Mohamed, Jianzhong Sun, Sameh S. Ali

**Affiliations:** ^1^Biology Department, College of Science, Taif University, Taif 21944, Saudi Arabia; ^2^Botany and Microbiology Department, Faculty of Science, Tanta University, Tanta 31527, Egypt; ^3^Department of Biotechnology, College of Science, Taif University, Taif 21974, Saudi Arabia; ^4^Department of Molecular Medicine, Princess Al-Jawhara Centre for Molecular Medicine, School of Medicine and Medical Sciences Arabian Gulf University, Manama 329, Bahrain; ^5^Pharmacology and Toxicology Unit, Department of Biochemistry, Animal Health Institute, Giza, Egypt; ^6^Biofuels Institute, School of the Environment and Safety Engineering, Jiangsu University, Zhenjiang 212013, China

**Keywords:** marine actinobacteria, silver nanoparticles, antimicrobial, insecticidal, antioxidant, cytotoxicity

## Abstract

Nanoparticles have recently emerged as a popular research topic. Because of their potential applications in therapeutic applications, biosynthesized silver nanoparticles (Bio-AgNPs) have gained much attention in recent years. Cell-free extracts (CFE) from a marine culture of actinobacteria and silver nitrate were used to reduce Ag^+^ ions and create Bio-AgNPs. *Nocardiopsis dasonvillei* KY772427, a new silver-tolerant actinomycete strain, was isolated from marine water and used to synthesize AgNPs. In order to characterize Bio-AgNPs, UV-Vis spectral analysis, Fourier transform infrared (FTIR), transmission electron microscopy (TEM), and dynamic light scattering spectroscopy (DLS) were all utilized. Using UV–Vis spectroscopy, a peak in the surface plasmon resonance (SPR) spectrum at 430 nm revealed the presence of Bio-AgNPs. The TEM revealed spherical AgNPs with a diameter of 29.28 nm. DLS determined that Bio-AgNPs have a diameter of 56.1 nm and a negative surface charge (−1.46 mV). The minimum inhibitory concentration (MIC) of Bio-AgNPs was determined against microbial strains. Using resazurin-based microtiter dilution, the synergistic effect of Bio-AgNPs with antimicrobials was investigated. *Pseudomonas aeruginosa* had the lowest MIC of Bio-AgNPs (4 μg/ml). Surprisingly, the combination of antimicrobials and Bio-AgNPs had a significant synergistic effect on the tested strains. The insecticidal activity of Bio-AgNPs (200 μg/ml) against *Macrosiphum rosae* was found to be maximal after 36 h. Additionally, Bio-AgNPs demonstrated significant scavenging activity against 2,2′-diphenyl-1-picrylhydrazyl (DPPH) and hydroxyl (OH**^–^**) radicals, with *IC*_50_ values of 4.08 and 8.9 g/ml, respectively. *In vitro* studies using the 3-(4,5-dimethylthiazol-2-yl)-2,5-diphenyl tetrazolium bromide (MTT) assay revealed a concentration-dependent decrease in cell viability when CaCo2 cells were exposed to Bio-AgNPs. With the decrease in cell viability, lactate dehydrogenase leakage (LDH) increased. The findings of this study open up a new avenue for the use of marine *Nocardiopsis dasonvillei* to produce Bio-AgNPs, which have significant antimicrobial, antioxidant, insecticidal, and anticancer potential.

## Introduction

Nanoparticles (materials with a dimension of less than 100 nm) are at the forefront of nanotechnology developments in biomedical research (Makvandi et al., 2020). Nanoparticles have a high surface area-to-volume ratio, which confers unique properties on them and enhances their catalytic, magnetic, mechanical, and optical properties, thereby expanding their potential biomedical applications (Ali et al., 2021; Nikzamir et al., 2021). Silver, among various metals, has long been recognized in applications, including food preservation, antimicrobial therapy, and water purification (Otari et al., 2015; Makvandi et al., 2019). Recent advancements in nanotechnology have resulted in the widespread use of silver nanoparticles (AgNPs) as antimicrobial, anti-inflammatory, and anti-cancerous agents (Khalil et al., 2021), since AgNPs have unique optical, magnetic, catalytic, and electronic properties (Sánchez-López et al., 2020).

Scientific research has shown that AgNPs can interfere with cellular functions *via* a variety of mechanisms. One of these mechanisms is the apoptosis activation (Senthil et al., 2017). AgNPs agglomerated in the nucleus, mitochondria, and lysosomes, cause significant oxidative damage in these organelles and ultimately lead to apoptosis or necrosis (Nikzamir et al., 2021). Another mechanism for AgNPs cytotoxicity is the production of reactive oxygen species (ROS) as a result of disruption of the mitochondrial electron transferring chain, which causes DNA damage (Dos Santos et al., 2014). The cytotoxicity of AgNPs has recently gained prominence as a research topic because of their ultra-small size that can easily enter cells and move through the bloodstream (Wei et al., 2010). As a result, smaller AgNPs (3–7 nm) are more cytotoxic to mouse cells than larger AgNPs (10–40 nm) (Liu et al., 2010). Moreover, AgNPs exert their antimicrobial effects by binding to microbial DNA, resulting in a DNA degradation (Nikzamir et al., 2021). Therefore, the potential of AgNPs for therapeutic and biotechnological applications has gained much attention in recent years.

For the synthesis of AgNPs, various physical and chemical methods such as microwave assistance, thermal decomposition, and chemical reduction have been employed. However, these conventional methods are energy intensive, use hazardous chemicals, are time consuming, and pose environmental and biological risks, precluding their therapeutic applications (Otari et al., 2015; Bahlol et al., 2019; Khalil et al., 2021). On the other hand, biological methods are simple, quick, and inexpensive approaches to synthesize nanostructure materials as well as to reduce the generation of hazardous substances toxic to the environment and human health (Singh et al., 2020; Nikzamir et al., 2021). Therefore, the emphasis for nanoparticle synthesis is shifting away from the conventional methods (physical and chemical) toward “green” chemistry, i.e., biological synthesis. Microorganisms almost certainly contribute to the production of a large number of nucleation centers and to the establishment of conditions conducive to the formation of highly dispersed nanoparticle systems. In addition, microorganisms immobilize the particles and provide a viscous medium containing extracellular secreted enzymes to completely prevent or minimize the aggregation rate (Bahlol et al., 2019). Several studies have been published on the use of bacteria such as *Bacillus subtilis*, as well as fungi such as *Fusarium oxysporum*, in the biosynthesis of AgNPs (Hulkoti and Taranath, 2014).

Actinobacteria are ubiquitous and most versatile Gram-positive bacteria with a high GC content. Enzymes produced by actinobacteria hold many great industrial applications. Furthermore, secondary metabolites secreted by actinobacteria enhance the synthesis of AgNPs by reducing activity (Manimaran and Kannabiran, 2017). The bioactive compounds produced by actinobacteria have various therapeutic antimicrobial, anticancer, and anthelmintics properties (Goel et al., 2021). *Nocardiopsis* sp. MBRC-1, a novel marine actinobacterium, biosynthesized AgNPs (Bio-AgNPs) with antimicrobial and cytotoxic properties (Manivasagan et al., 2013). Bio-AgNPs produced by *Streptomyces xinghaiensis* OF1 revealed potential antimicrobial activities against pathogenic bacteria and yeasts (Wypij et al., 2018). AgNPs’ physicochemical properties are critical to their cytotoxicity. As a result, it is thought necessary to assess the cytotoxicity of AgNPs in order to maintain a safe level of silver in living organisms, as well as to comprehend how AgNPs interact with biological cells in order to gain a better understanding of the health risks associated with the use of nanoparticles (Liao et al., 2019). On the other hand, tobacco cutworm, for example, is a well-known cosmopolitan pest of a variety of crops. This voracious pest’s survival is aided by the frequent evolution of insecticide resistance and its polyphagous nature. Nanotechnology provides an alternative approach for overcoming the limitations of current pest management strategies (Jafir et al., 2021). For their potential use in crop protection, AgNPs have attracted the attention of entomologists due to least toxicity to the environment and humans.

Multidrug resistance (MDR) is a persistent global issue. A considerable need for revolutionary nanomaterials has developed for the construction of new antimicrobial biomaterials, which has unavoidably opened up new therapeutic horizons in medical approaches (Khalil M. A. et al., 2015; El-Zawawy and Ali, 2016; Khalil et al., 2020, 2021). To the best of our knowledge, no studies have been published on the efficacy of Bio-AgNPs produced by the marine actinobacterium *Nocardiopsis dassonvillei* as an antimicrobial candidate against MDR pathogens with concurrent antioxidant, insecticidal, and anticancer potential. Under this scope, this study aims at exploring therapeutic properties of Bio-AgNPs using cell-free extract (CFE) of *N. dassonvillei* actinobacterium. The study’s findings open a new path for the production of Bio-AgNPs with strong antibacterial, antioxidant, insecticidal, and anticancer potential from marine *N. dasonvillei*.

## Materials and Methods

### Samples and Microbial Strains

Marine water and sediment samples were collected from five different locations; the north beach Jeddah, Southern beach in Jeddah, Abhor, Laith, and Qunfudah (21^⋅^38′54″ North-39^⋅^06′06″ East, 21^⋅^19′44″ North-39^⋅^06′33″ East, 21^⋅^45′02″ North-39^⋅^08′01″ East, 20^⋅^49′38″ North-39^⋅^25′15″ East, 19^⋅^11′36″ North-41^⋅^02′40″ East) on the Red Sea coast, Jeddah, KSA. Sediment samples (3–5 cm depth) were collected through a sterile pustule and placed in sterile plastic bags. Marine water samples were taken in a sterile bottle at a depth of 10 cm. For marine actinobacteria isolation, all samples were securely sealed, put in ice containers, and promptly transported to the Microbiology Laboratory, Faculty of Science, Taif University, KSA.

For antimicrobial activity test, seven representative bacterial isolates, namely *Staphylococcus aureus*, coagulase-negative (CoNs) *Staphylococcus*, *Pseudomonas aeruginosa*, extended spectrum beta-lactamase (ESβL)-producing *Escherichia coli*, *Salmonella* sp., *Klebsiella pneumoniae*, and *Proteus mirabilis* and two fungal species (*Aspergillus niger* and *Candida albicans*). Both bacterial and yeast cultures were maintained in 10% glycerol stock at −80°C. The filamentous fungus, *A. niger*, was preserved in potato dextrose agar (PDA) and stored in 10% glycerol stock at −80°C.

### Isolation of Marine Actinobacteria and Screening for Tolerance to Silver Nitrate

**FIGURE 1:**
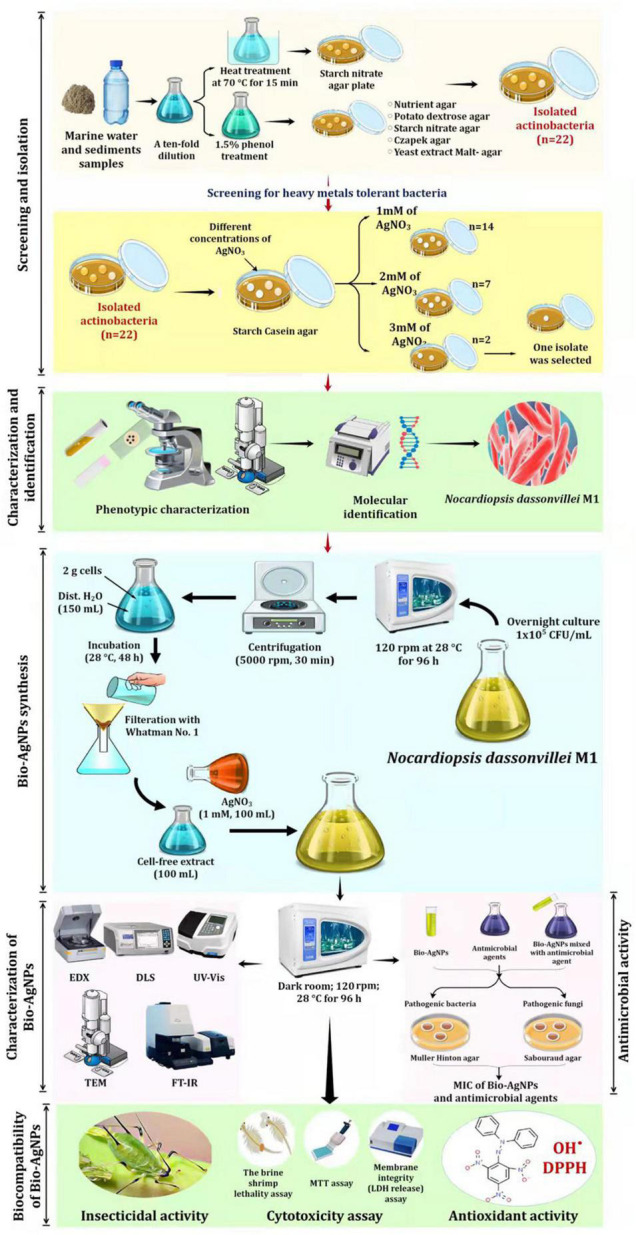
Experimental set up for biosynthesis of silver nanoparticles by marine actinobacterium *Nocardiopsis dassonvillei* M1 and exploring their therapeutic potentials.

Figure 1 depicts the experimental setup used to screen and isolate actinobacteria capable of tolerating silver nitrate (AgNO_3_) from marine water. Actinobacteria were isolated from marine water and sediment samples using the spread plate technique (Manivasagan et al., 2013). To isolate actinomycetes, all samples were heated in a water bath at 70°C for 15 min and treated with 1.5% phenol, then cultured on starch nitrate agar medium at 28°C for 14 days. To reduce nitrates, isolates were cultured for 7 days in nitrate-containing medium before being reduced with the Griess-Illosvays reagent (Dixit et al., 2015). From the collected samples, 110 isolates were obtained and cultured for 7 days at 28°C on various media, including yeast extract-malt agar, Czapek agar, nutritional agar, and potato dextrose agar (Figure 1). Twenty-two colonies with typical actinobacteria characteristics were subcultured on starch casein agar (SCA) medium supplemented with different concentrations of AgNO_3_ to assess their metal resistance tolerance. As a result, the metal-resistant isolate designated as M1 was chosen for the production of Bio-AgNPs. Stock bacterial cultures were kept at −80°C in 0.05 M potassium sodium phosphate buffer (pH 7.0), containing 10% glycerol (v/v).

### Molecular Characterization

The chosen isolate M1 was characterized molecularly based on 16S rDNA gene sequencing as described by Ali et al. (2020a). The total genomic DNA of bacteria was extracted using the TaKaRa extraction kit (TakaRa, Japan) according to the manufacturer’s instructions. A pair of universal bacterial primers 27 F (5′-AGAGTTTGATCCTGGCTCAG-3′) and 1492R (5′-TACGGCTACCTTGTT ACGACTT-3′) was used for the amplification step. The PCR amplification was performed as described previously (Ali et al., 2020b). The purified amplification products were sequenced at Macrogen Co., Seoul, South Korea. The sequences were then deposited in the GeneBank^1^. The sequence homology of M1 with the closest similar bacterial strains was determined using a BLAST search (see text footnote 1). MEGA 7.0 software was used to construct a phylogenetic tree (Kumar et al., 2016).

### Preparation of Biosynthesized Silver Nanoparticles

As shown in Figure 1, Bio-AgNPs were synthesized using AgNO_3_ (Sigma Aldrich) as a precursor and the actinobacterium’s secondary metabolites as reducing agents. An aliquot (20 μl) of the overnight *N. dasonvillei* M1 culture (1 × 10^5^ CFU/ml) was inoculated in 100 ml nutrient broth medium and incubated on an orbital shaker at 28°C for 96 h at 120 rpm. Actinobacterium biomass was collected after incubation by centrifugation at 5000 rpm for 30 min. To remove the associated medium components, 2 g of cells were washed twice with distilled water. After that, the biomass was resuspended in 150 ml of distilled water and stored at 28°C for 48 h. After osmotically lysing the bacteria, they were filtered through a Whatman No. 1 filter paper, yielding CFE, which was used as a reducing agent in the preparation of Bio-AgNPs. The pH, temperature, and AgNO_3_ concentration were all adjusted for the synthesis of Bio-AgNPs (Veerasamy et al., 2011).

### Characterization of Biosynthesized Silver Nanoparticles

To characterize Bio-AgNPs, the optimal reaction mixture was created by combining CFE and AgNO_3_ solutions and incubating them for 96 h on an orbital shaker at 28°C (in the dark) as depicted in Figure 1. The color change of the reaction mixture from yellowish-white to dark brown indicated the formation of AgNPs (Siddiqi et al., 2018). The Bio-AgNPs was monitored using UV–visible (UV–Vis) spectroscopy (Shimadzu No-UV 1800) with a resolution of 1 nm in the 300–800 nm range. Fourier transform infrared spectroscopy (FTIR; PerkinElmer) was used to further characterize changes in the surface composition (Ali et al., 2022). The Bio-AgNPs were pelletized with potassium bromide (KBr) at a concentration of 1% (w/w), then pressed for 1 min at 10 t of pressure to form a transparent pellet, and FTIR analysis was determined between a transmittance range of 500 and 4000 cm^–1^. Energy Dispersive X-ray (EDX) was performed to identify the elemental composition of the AgNPs (Goel et al., 2021). Transmission electron microscopy (TEM; JEM-1230, JEOL, Japan) with a 120 kV acceleration voltage was used to determine the size, distribution, and morphology of the Bio-AgNPs (Khalil et al., 2021). The average size and surface charge of the Bio-AgNPs was determined using dynamic light scattering (DLS) (Goel et al., 2021). Before analysis, the Bio-AgNPs sample (1 mg/ml) was diluted 100 times in MiliQ water and ultrasonicated to ensure homogeneous distribution of nanoparticles and to prevent aggregation, if any. The sample was then analyzed using a Malvern DLS instrument (Nano-Zeta Sizer-HT, United Kingdom).

### Determination of Biosynthesized Silver Nanoparticles Stock Concentration

The concentration of the stock Bio-AgNPs colloidal suspension was calculated as per our earlier report (Khalil et al., 2021). A stock solution of Bio-AgNPs in dist. H_2_O with a final concentration of 1.7 μg/ml was prepared. To create working solutions, serial dilutions with a suitable culture medium were used.

### Antibiotic Susceptibility Test and Multidrug Resistance Determination

Antibiotic susceptibility test of bacteria and fungi was performed by disc diffusion method (CLSI, 2018) on Müller-Hinton (MHA) and Sabouraud’s dextrose (SDA) agar plates, respectively, as described previously (Ali et al., 2017). For inoculum preparation, bacterial and yeast isolates were cultured on nutrient agar and SDA and the plates were incubated for 18 h at 37°C. The cultures were then suspended in a sterile normal saline solution to achieve an inoculum final concentration of 1 × 10^5^ CFU/ml. For inoculum preparation of *A. niger*, the culture was maintained on SDA. After incubation for 7 days at 37°C, the conidial suspension was prepared to achieve a concentration of 1 × 10^5^ CFU/ml (Ali et al., 2017). To assess sensitivity, inoculated agar plates supplemented with the antibiotic discs tested (Table 1) were incubated for 18 h and the inhibition zone diameter (IZD) surrounding the discs was measured. The results were interpreted (CLSI, 2018) and resistance to at least three antimicrobial groups was defined as MDR (Khalil et al., 2021).

**TABLE 1:** Antibiotic resistance patterns among bacterial and fungal pathogens used in this study.

Microbial pathogen	Pattern profile
*Staphylococcus aureus*	AM, TZP, VA, PB
*Salmonella* sp.	TZP, CAZ, VA, TE, CMN, PB
*Klebsiella pneumoniae*	AM, TZP, CAZ, VA, CMN, PB
*Pseudomonas aeruginosa*	AM, AMC, TZP, SXT, VA, TE, CMN
CoNs *Staphylococcus*	TZP, NOR, CIP, CN, SXT, VA, TE, CMN, AK, PB
ESβL-producing *Escherichia coli*	AM, AMC, TZP, CTX, CAZ, FEP, NOR, CIP, CN, SXT, VA, TE, CMN, PB
*Proteus mirabilis*	AM, TZP, CTX, CAZ, FEP, NOR, CIP, CN, SXT, VA TE, CMN, AK, PB
*Aspergillus niger*	ITC, FLZ, NYT, TRB
*Candida albicans*	FLZ, AMP, NYT, MIZ

*AM, Ampicillin; AMC, Amoxicillin-clavulanate; TZP, Piperacillin-tazobactam; CTX, Cefotaxime; CAZ, Ceftazidime; FEP, Cefepime; NOR, Norfloxacin; IPM, Imipenem; CIP, Ciprofloxacin; CN, Gentamicin; SXT, Trimethoprim-sulfamethoxazole; VA, Vancomycin; TE, Tetracycline; CMN, Clindamycin; AK, Amikacin; PB, Polymyxin; ITC, Itraconazole; FLZ, Fluconazole; NYT, Nystatin; TRB, Terbinafine; AMB, Amphotericin; MIZ, Miconazole.*

### Antimicrobial Activity of Biosynthesized Silver Nanoparticles

As depicted in Figure 1, *in vitro* antimicrobial activity of Bio-AgNPs was performed using agar well diffusion methods (EUCAST, 2020). Inoculum and plate preparations were carried out as mentioned above. Different concentrations of Bio-AgNPs (50, 100, 150, and 200 μg/ml) were prepared, and 50 μl of each concentration was added to every well. After incubation for 18 h, IZD surrounding each well was determined. CFE and AgNO_3_ were used as controls. The antimicrobial activity of piperacillin-tazobactam (TZP) alone, fluconazole (FLZ) alone, and their combinations with well as the synergistic effects of these antibiotics in combination with Bio-AgNPs were investigated against MDR isolates.

Minimum inhibitory concentration (MIC) of Bio-AgNPs was performed using resazurin-based microtiter dilution technique (CLSI, 2018). The Bio-AgNPs working solutions were made by diluting the stock colloidal solution (1.7 g/ml) with sterile dist. H_2_O. Bio-AgNP concentrations were varied between 0.002 and 0.5 μg/ml. A volume of 100 μl (1 × 10^5^ CFU/ml) of tested microorganisms was inoculated in the 96-well microtiter plates loaded with the prepared Bio-AgNPs concentrations and all plates were then incubated for 18 h at 37°C. Following incubation, each well received 20 μl of resazurin dye (0.1% w/v in dist. H_2_O) and the plates were kept at 37°C for 1 h. The color shift from purple to red indicated that cells were actively metabolizing, whereas the presence of dark blue indicated that microorganisms were completely prevented from growing in microtiter plate wells. At 600 nm, a Microplate Reader (Biorad, United States) was used to detect microbial growth or suppression. On the other hand, using the resazurin microdilution technique, the sensitivity of bacteria to TZP and fungi to FLZ was also determined (CLSI, 2008). To perform twofold serial dilutions of antibiotics, the stock concentrations were diluted with sterile H_2_O to determine the MIC of the antibiotic alone or in combination with the Bio-AgNPs. The experiment was carried out in triplicate on 96-well microtiter plates, and antibiotic concentrations ranging from 0.25 to 128 μg/ml were tested. The ultimate microbial density in each well was 1 × 10^5^ CFU/ml. MIC was the lowest concentration of Bio-AgNPs at which no visible growth was observed.

### Insecticidal Activity of Biosynthesized Silver Nanoparticles

The insecticide activity of Bio-AgNPs was evaluated using the plant dip technique established by Bhattacharyya et al. (2016). An insect colony of *Macrosiphum rosae* was grown on potted rose plants at 22 ± 2°C at the Entomology Laboratory, Department of Zoology, Taif University, KSA. Five leaves of *Rosa damascene* var *semperflorens* were dipped in various concentrations of Bio-AgNPs solutions (50, 100, 150, 200 μg/ml) or a control solution (water) for 15 s and left to dry at room temperature. Transfer the leaves to a plastic container and then gently place the insects on the leaves for feeding, allowing *M. rosae* to feed on both treated and untreated leaves. Three repetitions were repeated for each concentration. After 8, 16, 24, 36, 40, and 48 h of treatment, the proportion of insects that died was determined.

### Antioxidant Activity of Biosynthesized Silver Nanoparticles

The antioxidant activity of Bio-AgNPs was assessed in terms of free radical scavenging activity (RSA) using 1,1-diphenyl-2-pyridyl-hydrazine (DPPH) assay (Singh et al., 2018) and hydroxyl radical (OH^–^) scavenging activity method (Tanamatayarat, 2016). RSA of Bio-AgNPs was determined using the 1,1-diphenyl-2-pyridyl-hydrazine (DPPH) assay. Using DPPH assay, 4 mg of 0.02 mM DPPH was dissolved in 100 ml of methanol and kept at 4°C until needed. A 2 ml stock solution was added to 1 ml methanol containing various concentrations of test Bio-AgNPs (6.25–200 μg/ml). Methanol and CFE were used as negative controls in this experiment, while ascorbic acid was used as a positive control. After 30 min of incubation in dark conditions, all mixtures were measured at 517 nm using a UV–visible spectrophotometer (Shimadzu-UV2600, Japan). When a single electron of DPPH is paired with a hydrogen atom from a potential antioxidant, the color changes from purple to yellow. The OH^–^ RSA of the Bio-AgNPs was carried out using a range of concentrations (6.25–200 μg/ml) of the investigated Bio-AgNPs, as well as ascorbic acid as a standard. The antioxidant activity by DPPH and OH^–^ radical was calculated using the formula: % RSA = (control absorbance - sample absorbance/control absorbance) × 100. The linear regression curve was used to calculate the concentration of Bio-AgNPs needed to scavenge 50% of the radicals (*IC*_50_).

### Cytotoxicity Assay

Human colorectal adenocarcinoma (CaCo2) cells (5 × 10^4^ cells/ml) were grown in DMEM medium supplemented with 2 mM L-glutamine, 10% fetal calf serum (FCS), and 1% penicillin-streptomycin solution, then incubated for 24 h at 37°C in a humidified environment of 5% CO_2_ and 95% air. The medium was removed and replaced with a new medium after being rinsed with 0.1 ml of phosphate saline buffer (PSB). Cells were allowed to adhere to the surface for 24 h prior to treatment. Then, the culture medium in each well was replaced with a new medium containing Bio-AgNPs at concentrations ranging from 6.25 to 200 μg/ml. The cells were harvested after 24 h of incubation and assessed for viability and lactate dehydrogenase (LDH) release (Gurunathan et al., 2018).

Cell viability was calculated by the 3-(4, 5-dimethyl-2-thiazolyl)-2, 5-diphenyl-2H-tetrazolium bromide (MTT) colorimetric assay following Chen et al. (2006). In 96-well tissue culture plates, CaCo2 cells were seeded with varying amounts of Bio-AgNPs. All cultures were incubated for 24 h at 37°C in a humidified environment. Following incubation, 0.02 ml of MTT solution (5 mg/ml in PBS) was added to each well. The plates were then incubated for 4 h at 37°C in a humidified atmosphere of 5% CO_2_. The formazan (product of MTT reduction) was dissolved in 150 μl of dimethyl sulfoxide (DMSO) at 37°C with moderate shaking, and the absorbance at 490 nm was measured with a BIORAD microplate ELISA reader. Cells that had not been treated with Bio-AgNPs were used as a control. Cell viability percentage (% CV) was calculated using the formula: % CV = (absorbance of the experimental group/absorbance of control group) * 100. On the X-axis, the concentration of AgNPs was plotted against the % CV on the Y-axis, yielding the *IC*_50_ value.

The cell membrane integrity of CaCo2 cells was determined using the LDH activity colorimetric assay kit (K726-500) according to the manufacturer’s instructions (Biovision, Mountain View, CA). The amount of LDH that leaked out of the cell determined the integrity of the plasma membrane of Bio-AgNPs-treated cells. Following a 24 h treatment with different concentrations of Bio-AgNPs, 100 μl of each cell-free supernatant was added in triplicate to wells in a 96-microtiter plate, followed by 100 μl of LDH-assay reaction mixture in each well. The optical density at 490 nm was determined using a microplate reader after 3 h of incubation under standard conditions.

The brine shrimp lethality test was used to assess the *in vitro* cytotoxicity of Bio-AgNPs (Kummara et al., 2016). *Artemia salina* eggs were incubated in 1000 ml sterile saltwater with continuous aeration for 48 h. After hatching, collect 10 larvae and place them in a glass vial filled with 4.5 ml sterile saltwater. Then, 0.5 ml of Bio-AgNPs at various concentrations was added to the nauplius in each vial. Under light conditions, all vials were incubated at room temperature for 24 h. The number of living nauplii in each container was counted after 24 h of treatment, and the percentage of brine shrimp nauplii mortality was calculated. Based on this value, calculate the *LC*_50_ of the sample.

### Statistical Analysis

The data was interpreted using GraphPad Prism version 9.0.0 and Minitab statistical software (19.2020.1, Minitab Inc., United States). Using one-way analysis of variance (ANOVA) with Tukey-Kramer multiple comparisons, the antimicrobial activity of antimicrobial agents against various pathogenic microorganisms and the mortality rate were compared. The scavenging activities of the OH and DPPH radicals were investigated using an unpaired *t*-test. Data were expressed as the mean ± standard deviation (SD) from three replicates. The *P*-value of <0.05 denotes statistical significance.

## Results and Discussion

**FIGURE 2:**
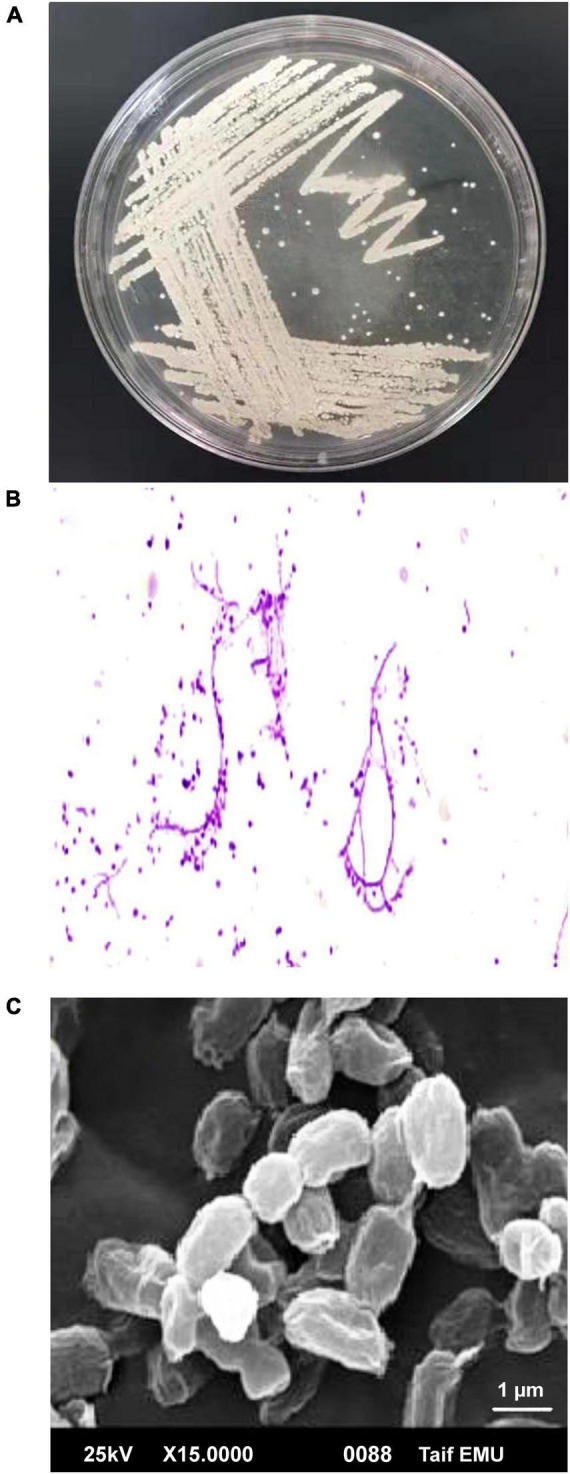
Morphological characteristics of *Nocardiopsis dassonvillei* M1. **(A)** Colonies on PDA plates. **(B)** Light microscopic image of hyphae with magnification 40X. **(C)** Scanning electron microscope of spores.

**FIGURE 3:**
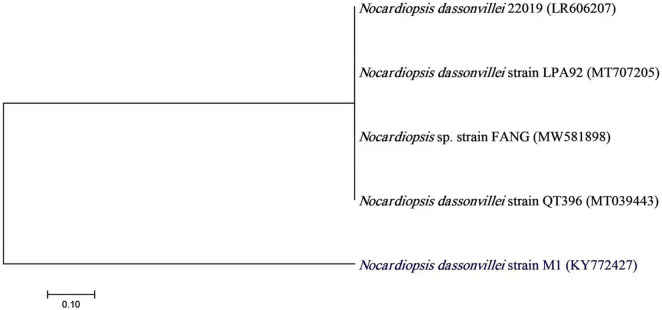
A neighbor-joining tree of *Nocardiopsis dassonvillei* M1 with its closely related taxa. The bootstrap consensus tree inferred from 1000 replicates represents the evolutionary history of the taxa analyzed. The scale bar indicates 0.10 substitutions per nucleotide position.

Actinobacteria, one of the many microbial groups found in the marine environment, occupy a significant niche as a potential source of novel metabolites and bioactive substances (Ganesan et al., 2017). In the current study, 22 out of 110 marine bacterial isolates exhibit conventionally distinct actinobacterial characteristics, such as the development of vegetative mycelium and a secondary aerial mycelium in permanent contact with the air, as well as the presence of a soluble pigment on various culture media (data not shown). The preliminary screening findings for heavy metal tolerant isolates revealed that 14 (63.63%) actinomycete isolates were tolerant to 1 mM Ag^+^, 7 (31.8%) isolates were tolerant to 2 mM, and only 2 (9.1%) isolates were tolerant to 3 mM. Therefore, for the preparation of Bio-AgNPs, the most tolerant isolate designated as M1 strain was chosen. Depending on the medium used, the color of the strain M1 mycelium substratum ranged from white to beige, while the color of the aerial mycelium ranged from white to gray, dark gray, and yellowish white. Figure 2 depicts the morphological characteristics of M1 strain. Figure 2A depicts white substrates and aerial mycelium in M1 colonies growing on PDA medium. Light and scanning microscopy images show that the sporangia of strain M1 have an irregular structure (Figures 2B,C). The isolate MW15, namely *N. dassonvillei* strain M1, was successfully identified molecularly (Figure 3). The 16S rDNA gene was amplified, and the sequence was submitted to GeneBank under the accession number KY772427^2^. BLAST analysis and the closest phylogenetic relative to the *N. dassonvillei* strain M1 exhibited high identity (100%) to *N. dassonvillei* strain QT396 (with accession number MT039443).

**FIGURE 4:**
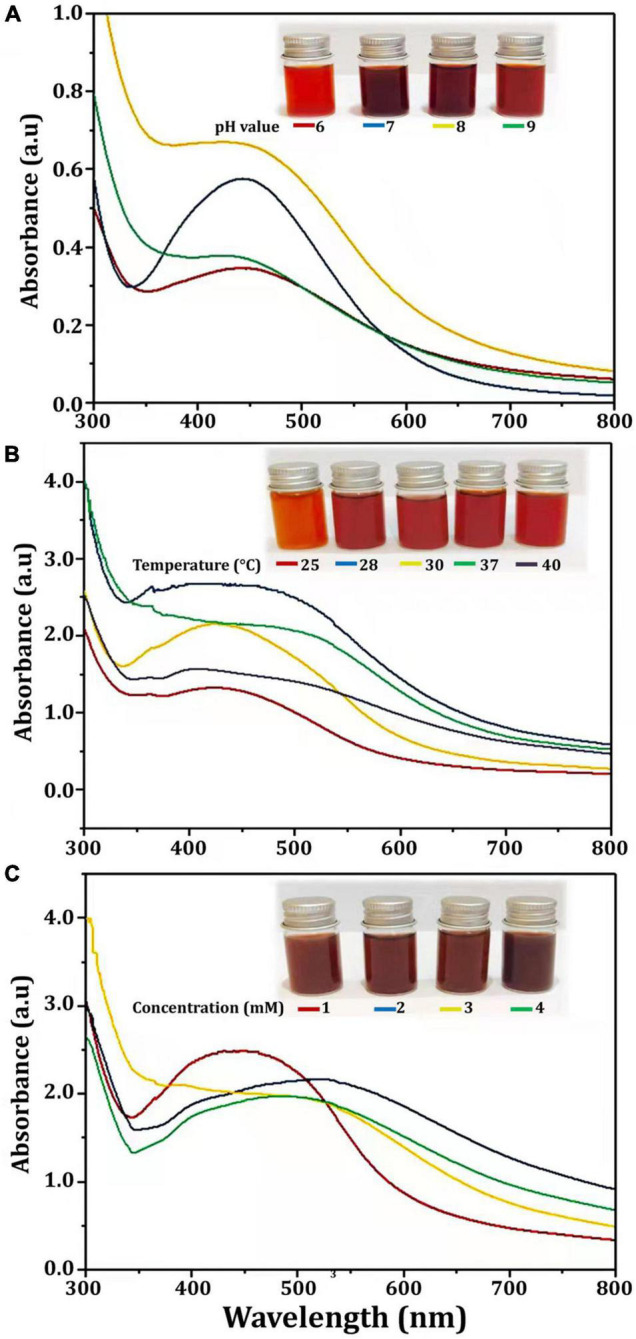
UV-visible spectra of Bio-AgNPs obtained at different reaction conditions. **(A)** Different *pH* value of reaction mixture, **(B)** Different reaction temperature (°C), **(C)** Different concentration of AgNO_3_ (mM).

Several physicochemical parameters (temperature, reaction mixture pH, and AgNO_3_ concentration) were optimized after being identified as variables influencing AgNPs yield (Veerasamy et al., 2011). With the addition of AgNO_3_, CFE was transformed into a brown yellow color, indicating the formation of AgNPs. This color is a result of the SPR of Bio-AgNPs. The conversion of silver ions to Bio-AgNPs was observed in spectrum data collected with a UV-Vis spectrophotometer. It has been reported that changing the pH of the reaction mixture changes the shape and size of the nanoparticles because pH changes the charge of biomolecules, which may affect their capping and stabilizing properties (Verma and Mehata, 2016). Clearly, the acidic pH (*pH* = 5) inhibited the production of Bio-AgNPs, but the intensity of the color increased as the pH solution increases from 6 to 9, which is attributed to the excitation of metal nanoparticle SPR (Figure 4A). Bio-AgNPs synthesized at *pH* = 7 exhibited a clear SPR peak at λ max 443 nm with absorbance 0.5769 a.u. Silver ion absorption occurred between 430 and 440 nm, according to SPR analysis (Chung et al., 2016). Veerasamy et al. (2011) reported that nanoparticles formed at pH 4, whereas tiny and highly dispersed nanoparticles formed at pH 8. According to Verma and Mehata (2016), the absorbance of AgNPs increases from 2 to 8 as the pH of the solution increases; as the pH of the solution increases further, the absorbance decreases. As illustrated in Figure 4B, as the temperature was increased, the absorbance peak was reached, indicating an increase in the rate of Bio-AgNPs synthesis, as indicated by the rapid change in the color of the reaction mixture. The UV-Vis spectrum at 28°C revealed a strong absorption SPR peak at 442.5 nm with an absorbance of 2.6833 a.u. However, absorbance was not increased at temperatures greater than 40°C. These findings are in accordance with that reported previously (Veerasamy et al., 2011; Singh et al., 2020). To obtain the optimal size of Bio-AgNPs, several AgNO_3_ concentrations were tested (Figure 4C). Increases in the concentration of AgNO_3_ in the reaction mixture result in the formation of additional Bio-AgNPs until a maximum yield of 1 mM AgNO_3_ with an absorption wavelength of 449 nm is achieved. Beyond 1 mM AgNO_3_, there was no discernible increase in absorbance. According to Veerasamy et al. (2011), a 5 mM concentration of AgNO_3_ promoted rapid production, while a 10 and 3 mM concentration of AgNO_3_ shifted the UV–visible spectrophotometer peak in plant extracts. In general, the results indicate that incubating the CFE (pH 7) and AgNO_3_ (1 mM) solution at 28°C on an orbital shaker for 96 h in the dark was the optimal reaction condition.

**FIGURE 5:**
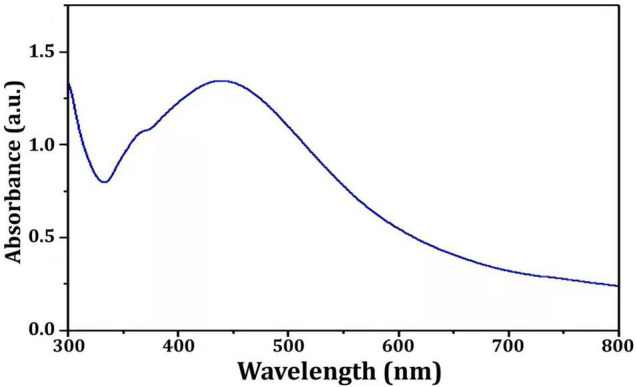
UV-visible spectra of the *Nocardiopsis dassonvillei* M1-derived Bio-AgNPs.

In this study, Bio-AgNPs were prepared using the CFE of the *N. dassonvillei* M1, which changed color from colorless to dark brown when treated with 1 mM AgNO_3_. UV–Vis spectroscopy was used to analyze the Bio-AgNPs and revealed a strong narrow SPR peak at 430 nm (Figure 5), which is attributed to their SPR property. It is most likely caused by the stimulation of longitudinal plasmon vibrations. Furthermore, the SPR property is responsible for the reaction mixture’s yellowish to brown color change. This could be due to secondary metabolites found in cells reducing metal ions (Manivasagan et al., 2013). The UV–Vis spectra confirmed the nanoparticle production from *Euphrasia officinalis* aqueous extract, as the absorption band for AgNPs was detected at 450 nm (Singh et al., 2018). Furthermore, numerous studies revealed that Bio-AgNPs were mostly formed using UV-vis-spectroscopy, with peaks ranging from 420 to 450 nm (Manivasagan et al., 2013; Singh et al., 2018).

**FIGURE 6:**
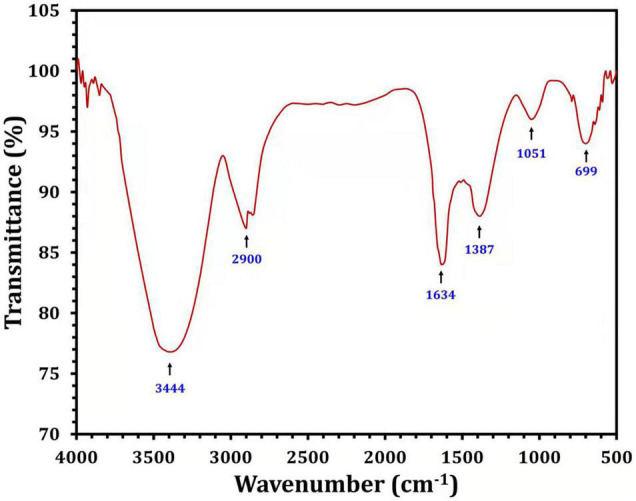
Fourier transform infrared spectra of the *Nocardiopsis dassonvillei* M1-derived Bio-AgNPs.

Fourier transform infrared analysis was used to identify the biomolecules responsible for the bioreduction of silver ions (Ag^+^) into AgNPs (Ag^0^) and the capping of the produced Bio-AgNPs. Several peaks have been detected as shown in Figure 6. The peak at 3444 cm^–1^ is assigned to the stretching vibrations of the O-H bonds of alcohols or phenols (Thirunavoukkarasu et al., 2013). The band between 2920 and 2850 cm^–1^ (C–H stretch) is assigned to the alkanes group, while the peak at 1634 cm^–1^ is assigned to the N-H bend of primary amines (Seyedeh et al., 2012). The peak at 1387 cm^–1^ is assigned to the symmetrical carboxyl group stretching (Kora et al., 2012). The OH deformation of alcoholic and phenolic OH groups is mediated by the peak at 1066 cm^–1^, while the peak at 699 cm^–1^ is assigned to the aromatic C-H (Litvin and Minaev, 2013). FTIR results clearly revealed the presence of phenolic compounds and proteins that are likely involved in the Bio-AgNPs, as well as the possibility that proteins play an important role in the stabilization of Bio-AgNPs by capping, which prevents agglomeration and helps to increase the stability of the Bio-AgNPs (Saranyaadevi et al., 2014).

**FIGURE 7:**
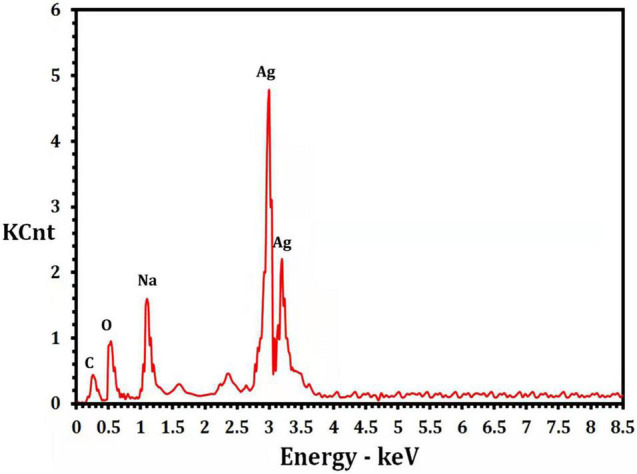
Energy dispersive x-ray analysis of the *Nocardiopsis dassonvillei* M1-derived Bio-AgNPs.

**FIGURE 8:**
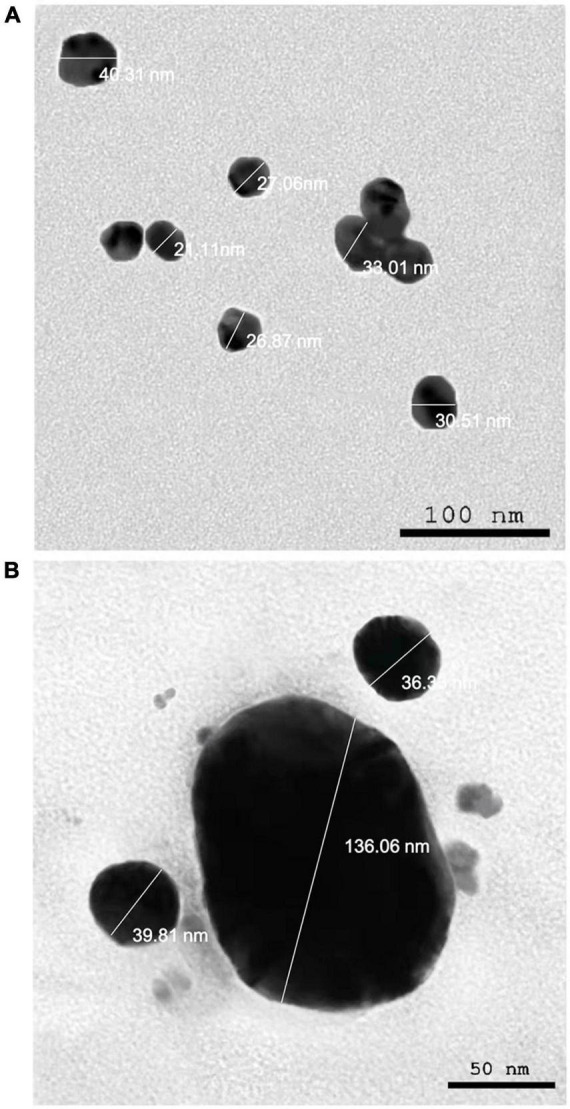
Transmission electron microscopy images of the *Nocardiopsis dassonvillei* M1-derived Bio-AgNPs. **(A)** In aqueous solution. **(B)** In DMEM culture medium.

The presence of elemental silver particles is demonstrated by the EDX graph (Figure 7). The chemical purity and existence signal of the elemental silver particles were validated using EDX analysis. The elemental composition analysis performed with EDX revealed a strong and sharp signal of silver atoms produced, with silver nanocrystals formed at an optical absorption band peak around 3 KeV, which is typical for metallic AgNPs absorption (Park et al., 2007), confirming the presence of AgNPs in the CFE (Figure 7). The obtained results are highly related to the TEM results (Figure 8). Other EDX signals were detected from O, Na, and C atoms, which correspond to X-ray emission from proteins present in cell-free filtrates and capable of attaching to nanoparticles *via* free amino groups or cysteine residues (Das et al., 2010). TEM was used to collect critical information about the size and morphology of primary nanoparticles. The average size of Bio-AgNPs in water and DMEM culture medium, as determined by TEM, was 29.28 ± 2.2 and 32.13 ± 3.4 nm, respectively (Figures 8A,B). In addition, the TEM images confirmed that the Bio-AgNPs were synthesized on a nanoscale, with most of them being monodispersed and of spherical shape (Figures 8A,B).

**FIGURE 9:**
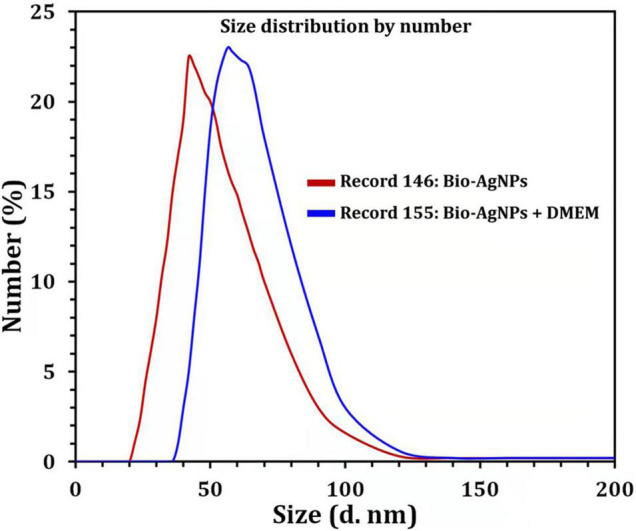
Size distribution by number graph of Bio-AgNPs. In aqueous solution (red line), in DMEM culture medium (blue line) as revealed by DLS.

Due to the fact that TEM images are obtained using a dry sample and a high vacuum, supplemental experiments utilizing DLS were done to evaluate the particle size in aqueous or physiological solutions. Prior to assessing *in vitro* toxicity, it is necessary to characterize nanoparticles in solution (Bin-Jumah et al., 2020). DLS and Zeta could be utilized to estimate the particle size and possible stability of AgNPs in aqueous solution and Dulbecco’s Modified Eagle Medium (DMEM) culture media, respectively. DMEM is the most broadly suitable medium for many adherent cell phenotypes among defined media for cell and tissue culture. As shown in Figure 9, DLS analysis revealed that the particle size of Bio-AgNPs in aqueous solution and DMEM was 56.1 and 64.7 nm, respectively, which is slightly bigger than the particle size detected in TEM, presumably due to Brownian motion. When Bio-AgNPs were exposed to DMEM medium, the proteins in the medium adsorb to the silver’s surface, forming a proteinous corona around the metal particles and increasing their “as dosed” size more than their “as prepared” size in water. As a result, despite the fact that Bio-AgNPs aggregate, the nanoparticles did not agglomeration due to their encapsulation in an organic layer. The net negative charges (zeta potential) on the surface of the evaluated Bio-AgNPs in aqueous solution and DMEM were enhanced from −1.46 to −6.81 mV, respectively. The high stability of AgNPs is essential for their biological applications. In cellular transport, particle size is crucial. The smaller the particle, the easier it is to get through the cell’s plasma membrane. As a result, it was believed that 100 nm nanoparticles were effective for a range of applications, including drug administration and the construction of biosensors (Elbeshehy et al., 2015; Khalil et al., 2017). Aside from size, the surface charge of AgNPs was assumed to be important for interaction with macromolecules and metabolic pathways. A strong negative zeta potential confers outstanding dispersity, colloidal nature, long-term stability, and no nanoparticle aggregation (Mohanta et al., 2017).

To test antibiotic susceptibility, the disc diffusion method was used, and the results are shown in Table 1. *E. coli* and *P. mirabilis* had the highest resistance patterns to the antibiotics tested. In general, all isolates exhibited an increased incidence of MDR (*n* = 4–14), with up to 50% of isolates exhibiting resistance to more than seven antibiotic agents (Khalil M. et al., 2015; Ali et al., 2017; Khalil et al., 2021). As illustrated in Table 1, the resistance profiles of the fungal isolates investigated were notably heterogeneous. Resistance to itraconazole (ITC), fluconazole (FLZ), nystatin (NYT), and terbinafine (TRB) was observed in *A. niger*. On the other hand, *C. albicans* revealed a resistant pattern, including amphotericin (AMB), miconazole (MIZ), FLZ, and NYT. Clearly, all pathogenic microorganisms exhibited phenotypic resistance to at least three distinct types of antibacterial and antifungal agents and were thus classified as MDR. Numerous studies demonstrated that microbes developed defensive mechanisms that made them more difficult to cure through the acquisition of resistance genes or genetic mutations, resulting in prolonged disease with a higher mortality rate (Ali et al., 2017; Khalil et al., 2021). Surprisingly, nanoparticles are being considered as a viable alternative to antibiotics and appear to hold significant promise for combating microbial MDR (Franci et al., 2015; Ali et al., 2021). As depicted in Table 1, pipercillin-tazobactam (TZP) was the most resistant antibacterial agent, whereas the FLZ was the most frequently resistant antifungal candidate. Therefore, TZP and FLZ were chosen for the *in vitro* antimicrobial activity test against selected isolates, either alone or in combination with Bio-AgNPs.

Antibiotic activity against microbial isolates was significantly enhanced when antibiotics were used in combination with Bio-AgNPs (Table 2). The IZD was observed using the agar well diffusion technique on MHA plates and two isolates were represented (Supplementary Figure 1). Bio-AgNPs have a significant inhibitory effect on tested isolates, with dose-dependent variation in sensitivity to Bio-AgNPs. The highest antibacterial activity was observed at 200 μg/ml for Bio-AgNPs or their combination with antibiotics. Bio-AgNPs demonstrated antibacterial activity at low concentrations (50 μg/ml), with an IZD ranging from 9.6 to 17 mm (*P* < 0.0001), whereas at high concentrations (200 μg/ml), the IZD ranged from 18.5 to 22.5 mm (*P* < 0.0001). These results corroborate previous research on the antimicrobial activity of AgNPs against *B. subtilis* and *C. albicans* (Wypij et al., 2018). The antibacterial efficacy of Bio-AgNPs-TPZ combination against selected clinical pathogens demonstrated the greatest synergism (Table 2). When TZP was combined with 200 μg/ml Bio-AgNPs, the greatest synergistic effect on *P. aeruginosa* and *Salmonella* sp. was observed, with an IZD of 29–26 mm, respectively (*P* < 0.0001). Additionally, Bio-AgNPs demonstrated potent antifungal activity at 200 μg/ml, and their activity was enhanced when combined with FLZ, a fungal growth inhibitor (Table 2).

**TABLE 2:** Performance of antibiotic alone, Bio-AgNPs alone, or their combination against various pathogenic microorganisms tested.

	Inhibition zone diameter (mm)								
Microorganisms	Antibiotic	Different concentrations of Bio-AgNPs (μg/ml) alone or in combination with selected antibiotics							
		50		100		150		200	
		Bio-AgNPs	Antibiotic + Bio-AgNPs	Bio-AgNPs	Antibiotic + Bio-AgNPs	Bio-AgNPs	Antibiotic + Bio-AgNPs	Bio-AgNPs	Antibiotic + Bio-AgNPs
	**TZP**								
*S. aureus*	17.0 ± 1.5^a^	10.0 ± 0.1^a^	17.5 ± 0.0^a^	12.5 ± 0.0^a^	18.5 ± 0.0^a^	15.0 ± 0.2^a^	19.8 ± 0.0^a^	18.5 ± 0.0^a^	19 ± 0.2 5^a^
CoNs *Staphylococcus*	19.0 ± 1.0^b^	11.0 ± 0.0^a^	20.0 ± 0.5^b^	13.0 ± 0.5^a^	21.5 ± 0.5^b^	15.0 ± 0.5^a^	21.7 ± 0.0^ab^	19.5 ± 0.0^ab^	22.2 ± 0.0^b^
*P. aeruginosa*	18.0 ± 2.0^ab^	11.0 ± 0.5^a^	19.5.0 ± 0^b^	15.5 ± 0.2^b^	23.0 ± 0.5^b^	17.0 ± 0.0^b^	26.0 ± 0.5^c^	20.8 ± 0.2^b^	29.0 ± 0.2^c^
ESβL-producing *E. coli*	18.5 ± 1.0^ab^	9.6 ± 0.2^a^	19.5 ± 0.2^ab^	12.4 ± 0.2^a^	21.5 ± 0.2^b^	15.0 ± 0.5^a^	22.5 ± 0.2^b^	21.5 ± 0.0^bc^	22.0 ± 0.0^b^
*Salmonella* sp.	20.0 ± 0.1^b^	13.5 ± 0.0^b^	21.0 ± 0.5^b^	15.5 ± 0.8^b^	23.5 ± 0.8^b^	17.0 ± 0.2^b^	24.5 ± 0.0^bc^	20.5 ± 0.3^b^	26.0 ± 0.0^d^
*K. pneumoniae*	19.0 ± 1.0^b^	17.0 ± 0.2^c^	19.5 ± 0.2^b^	19.0 ± 0.8^c^	20.0 ± 0.2^ab^	20.5 ± 0.5^c^	21.0 ± 0.0^ab^	22.5 ± 0.0^c^	24.0 ± 0.5^bd^
*P. mirabilis*	18.5 ± 1.0^ab^	14.0 ± 0.0^b^	19.7 ± 0.2^b^	16.5 ± 0.5^b^	21.0 ± 0.0^b^	17.5 ± 0.6^b^	23.0 ± 0.0^b^	19.0 ± 0.0^a^	24.8 ± 0.2^bd^
*P-* value	0.1557	<0.0001	<0.0001	<0.0001	<0.0001	<0.0001	0.0107	<0.0001	<0.0001
	**FLZ**								
*A. niger*	24.5 ± 0.20^a^	22.0 ± 0.2^a^	24.4 ± 0.25^a^	23.1 ± 0.5^a^	25.2 ± 0.25^a^	24.5 ± 0.25^a^	26.4 ± 0.25^a^	26.5 ± 0.2^a^	30.3 ± 0.2^a^
*C. albicans*	30.12 ± 1.0^b^	27 ± 0.2^b^	31.0 ± 0.3^b^	28 ± 0.5^b^	31.5 ± 0.2^b^	29 ± 0.2^b^	32.0 ± 0.2^b^	31 ± 0.2^b^	35.5 ± 0.2^b^
*P-*value	0.0008	<0.0001	<0.0001	0.0003	<0.0001	<0.0001	<0.0001	<0.0001	<0.0001

*The values are mean of three independent experiments. Values followed by the same letters showed insignificant difference. P-value of <0.05 denotes statistical significance replicates. TZP, Piperacillin-tazobactam; FLZ, Fluconazole.*

Biosynthesized silver nanoparticles colloidal stock solution was prepared, and the measured concentration was 1.7 μg/ml. Based on the MICs of Bio-AgNPs determined using a resazurin-based microtiter dilution assay (Table 3), all bacterial isolates were found to be highly sensitive to Bio-AgNPs, with MIC values ranging from 4 to 128 μg/ml. The obtained results showed that the TZP-MICs ranged from 8 to 128 μg/ml against the tested bacterial isolates. *P. aeruginosa* had the lowest MIC (4 μg/ml). Antibiotics combined with Bio-AgNPs were found to have a significant synergistic effect against tested isolates. When AgNPs and antibiotics were combined, Wypij et al. (2018) observed an increase in antibacterial activity against bacterial cells. This synergism resulted from a binding interaction between antibiotic molecules that contained hydroxyl and amino groups that could easily react with AgNPs. Antimicrobial activity is generally dose dependent, and it is more noticeable against Gram negative bacteria than Gram positive bacteria (Singh et al., 2018). Gram negative cells, which have a thin peptidoglycan layer, are presumably more susceptible to AgNPs permeation than Gram positive cells, which have a thicker peptidoglycan layer, forming an effective barrier against Bio-AgNPs penetration. These findings are in accordance with those reported previously (Rai et al., 2009). When Ag^+^ ions from AgNPs bind to the negatively charged cell surface, they alter the physical and chemical properties of cell membranes, impairing critical processes such as permeability, osmoregulation, electron transport, and respiration (Mohanta et al., 2017). AgNPs can inhibit the enzymatic activity of certain proteins by binding to the l-cysteine thiol groups (Rajeshkumar and Malarkodi, 2014). Furthermore, by permeating the cell and interacting with proteins, DNA, and other sulfur- and phosphorus-containing cell constituents, AgNPs can cause additional damage to bacterial cells (Nayak et al., 2015). As shown in Table 3, fungal isolates were found to be extremely sensitive to Bio-AgNPs, with MIC values of 32 μg/ml. FLZ-Bio-AgNPs combination had MICs of 8 and 16 μg/ml against *A. niger* and *C. albicans*, respectively, whereas FLZ alone had MICs of 64 and 128 μg/ml against *A. niger* and *C. albicans*, respectively. A fungicide effect of AgNPs on the evaluated fungal species could be explained by the nanoparticles acting simultaneously on multiple targets (Ishida et al., 2014).

**TABLE 3:** Minimum inhibitory concentration (MIC) of antibiotic alone, Bio-AgNPs alone, or their combination against various pathogenic microorganisms tested.

Microorganisms	MIC (μg/ml)		
	Antibiotic	Bio-AgNPs	Antibiotic + Bio-AgNPs
	**TZP**		
*S. aureus*	128	≤128	32
CoNs *Staphylococcus*	128	≥128	64
*P. aeruginosa*	4	≤8	0.5
ESβL-producing *E. coli*	64	16	4
*Salmonella* sp.	32	64	16
*K. pneumoniae*	64	<128	≤32
*P. mirabilis*	64	≤128	≤16
	**FLZ**		
*A. niger*	32	64	≤8
*C. albicans*	32	128	≤16

While numerous studies on the toxic effects of nanoparticles on bacterial and animal pathogens have been conducted (Rawtani et al., 2018), relatively little research has been conducted on the toxic effects of biosynthesized nanoparticles used to combat pest insects. To this end, the insecticidal activity of Bio-AgNPs was evaluated in this study. Bio-AgNPs synthesized by M1 strain have insecticidal activity against *M. rosae* found on *R. damascene* var *semperflorens* (Table 4). Bio-AgNPs had the highest mortality rate after 48 h, with the exception of 200 μg/ml, which had the highest mortality rate after 36 h. AgNPs were effective as insecticides at a concentration of 500 ppm (Bhattacharyya et al., 2016). The extraordinary strength, chemical reactivity, and electrical conductivity of nanoparticles may explain their remarkable efficacy as insecticides.

**TABLE 4:** Insecticidal activity of Bio-AgNPs.

Treatment (μg/ml)	Percentage mortality*					
	8 h	16 h	24 h	36 h	40 h	48 h
50	5.0 ± 0.5^a^	15.0 ± 0.5^a^	55.0 ± 0.5^a^	70.0 ± 0.0^a^	90.0 ± 0.5^a^	100.0 ± 1.1^a^
100	10.0 ± 0.5^be^	20.0 ± 1.1^be^	55.0 ± 1.1^b^	80.0 ± 0.5^be^	95.0 ± 0.5^ac^	100.0 ± 0.5^ac^
150	15.0 ± 0.5^cef^	30.0 ± 0.5^cef^	65.0 ± 1.1^ce^	90.0 ± 0.5^cef^	95.0 ± 0.5^bc^	100.0 ± 0.5^bc^
200	30.0 ± 0.5^df^	55 ± 0.5^df^	85.0 ± 0.5^de^	100.0 ± 0.5^df^	0.0 ± 0.0	0.0 ± 0.0

*Mean of three replications ± standard error; means followed by the same letters in each column are not significantly different by LSD at 5%.

**FIGURE 10:**
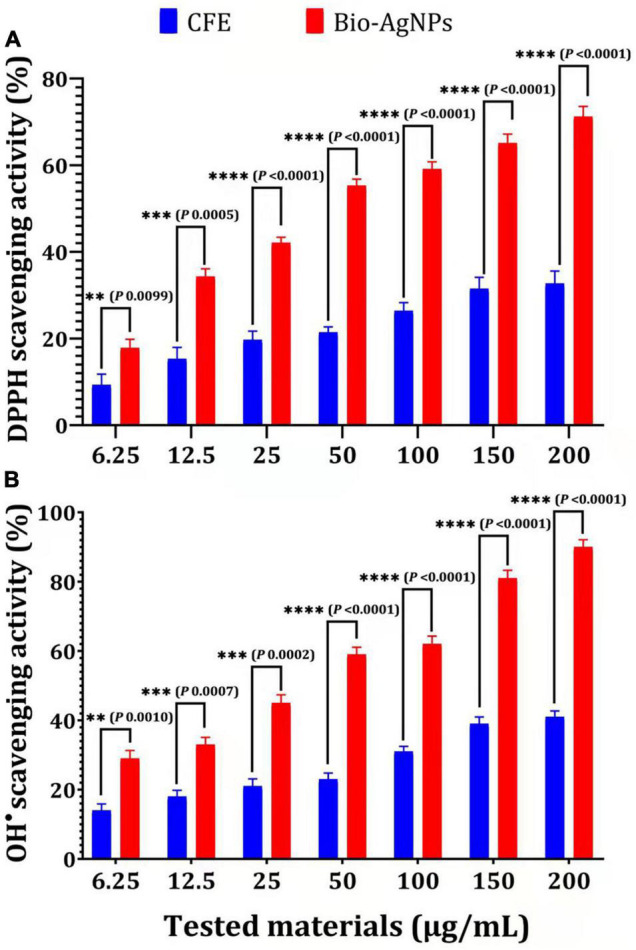
Antioxidant capacity of different concentrations of CFE and Bio-AgNPs. **(A)** DPPH radical scavenging activity. **(B)** OH^–^ radical scavenging activity. The data are represented in the form of a bar graph and plotted using mean ± SE of three replicates. *P*-values for significantly different mean values, *P* < 0.01 (^**^), *P* < 0.001 (^***^), *P* < 0.0001 (^****^).

Biosynthesized silver nanoparticles were evaluated *in vitro* for their antioxidant activity using the DPPH free radical scavenging method. The reduction of the DPPH radical to its non-radical form, DPPH–H, in the presence of a hydrogen-donating antioxidant is the basis for this technique. As illustrated in Figure 10A, the DPPH RSA (%) values of Bio-AgNPs increased in lockstep with the increasing Bio-AgNPs concentrations. The highest percentage of DPPH RSA activity (71.2 %) was obtained at the assay’s maximum concentration of 200 μg/ml, while the lowest activity (17.84 %) was obtained at 6.25 μg/ml. Bio-AgNPs were a powerful free radical scavenger when compared to CFE. Furthermore, when 200 μg/ml AgNPs were used instead of CFE, their antioxidant activity was increased by 2.17 times (*P* < 0.0001) as represented in Figure 10A. The *IC*_50_ values of CFE and Bio-AgNPs were determined to be 11.08 and 4.08 μg/ml, respectively. The DPPH assay is based on the reduction of DPPH in the presence of a hydrogen-donating antioxidant, which results in the formation of diphenylpicrylhydrazine. Sample compounds alter the color of DPPH due to their hydrogen-donating ability. The color change of the violet DPPH to yellow demonstrated Bio-AgNPs’ antioxidant activity (Singh et al., 2018). As depicted in Figure 10B, the Bio-AgNPs demonstrated significant OH^–^ radical scavenging activity, with a maximum scavenging activity of 90% at a concentration of 200 μg/ml, whereas the highest scavenging activity observed for CFE was 41% (*P* < 0.0001). The *IC*_50_ values of CFE and Bio-AgNPs were determined to be 8.9 and 3.33 μg/ml, respectively. The lower *IC*_50_ value of Bio-AgNPs shows their efficient free radical scavenging activity. Our findings indicate that the Bio-AgNPs exhibited extremely high antioxidant activity, suggesting their utilization in biomedical and pharmaceutical applications to protect against a variety of degenerative diseases associated with oxidative stress (Ahn et al., 2019).

**FIGURE 11:**
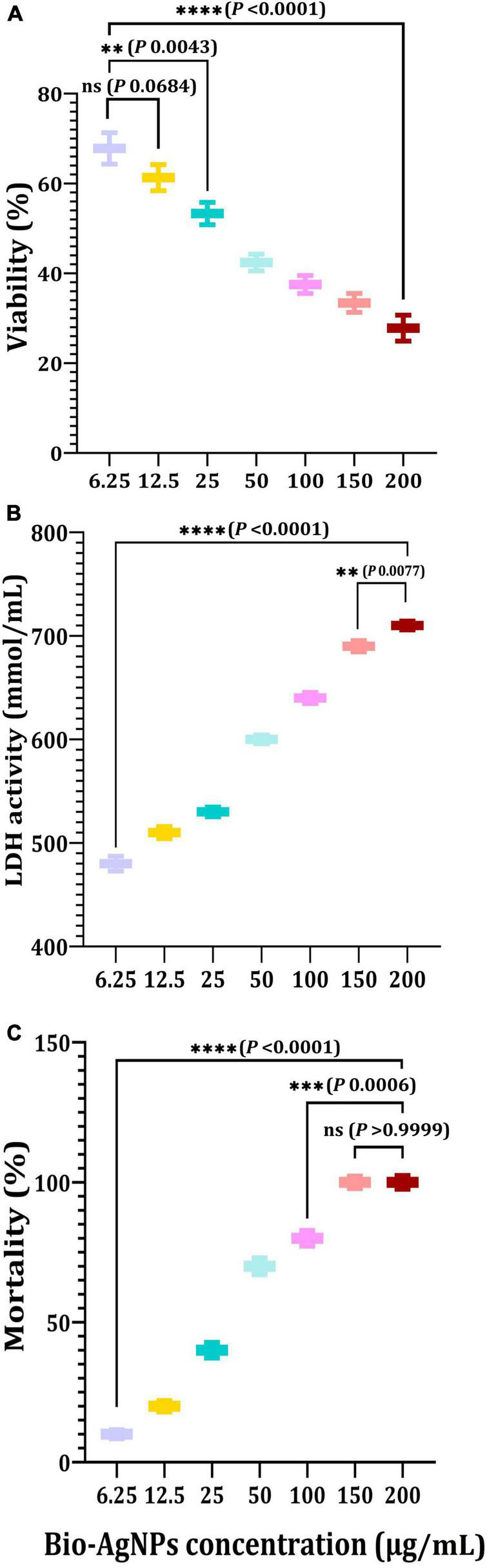
*In vitro* cytotoxicity of the *Nocardiopsis dassonvillei* M1-derived Bio-AgNPs against CaCo2 cell lines. **(A)** MTT assay. **(B)** LDH leakage assay. **(C)** Mortality of brine shrimp. The data are represented in the form of a bar graph and plotted using mean ± SE of three replicates. *P*-values for significantly different mean values, **ns**, non-significant, *P* < 0.01 (^**^), *P* < 0.001 (^***^), *P* < 0.0001 (^****^).

Cytotoxicity assessment represents a critical stage in the development of novel effective medicinal drugs (Bin-Jumah et al., 2020; Makvandi et al., 2021). The MTT assay was used in this study to determine the cytotoxicity of Bio-AgNPs on the CaCo2 cell line used as an *in vitro* model. As shown in Figure 11A, Bio-AgNPs have a dose-dependent effect on CaCo2 cell viability. The results showed that Bio-AgNPs at a concentration of 23.45 μg/ml reduced CaCo2 cell viability to 50%, which was determined to be the *IC*_50_. After 24 h of incubation, the *IC*_50_ of AgNPs on the CaCo2 cell line was determined to be 18 μg/ml (Zein et al., 2020). AgNPs have been shown to have a variety of cytotoxic effects in a variety of cell types, indicating that they harmed cell survival by disrupting mitochondrial structure and metabolism (Manivasagan et al., 2013; Składanowski et al., 2016). The LDH leakage test is a well-known cytotoxicity assay that measures cytotoxicity by detecting intracellular molecule leakage *via* damaged plasma membranes. LDH is a soluble cytoplasmic enzyme that is found in almost all cells and is released into the extracellular space when the plasma membrane is disrupted (Gurunathan et al., 2018). To identify LDH leakage into the cell’s exterior, cells were treated for 24 h with varying concentration of Bio-AgNPs (6.25–200 μg/ml), and leakage was quantified as the quantity of formazan amount generated using standard spectroscopy. As shown in Figure 11B, increased Bio-AgNPs dosages resulted in significantly increased cytotoxicity and LDH leakage (*P* < 0.0001), implying that cells exposed to Bio-AgNPs expand and lose membrane integrity before destroying and leaking their intracellular contents into the environment. The *IC*_50_ of Bio-AgNPs was determined to be 47.0 μg/ml, which had a significant effect on cell viability while also impairing cellular membrane integrity. These findings are in accordance with those reported by Gurunathan et al. (2018) who found that AgNPs stimulate colon cancer cells to release LDH. Brine shrimp bioassay lethality is an effective method for determining the cytotoxicity and broad range of pharmacological effects of bioactive chemicals (Priyaragini et al., 2013). As illustrated in Figure 11C, the brine shrimp lethality assay was used to further evaluate Bio-AgNPs’ cytotoxicity, as the effect of various Bio-AgNPs concentrations on *A. salina* brine shrimp mortality was significantly increased (*P* < 0.0001). After 24 h, AgNPs (50 μg/ml) exhibited a 70% inhibition, while 150–200 μg/ml exhibited a 100% inhibition. The *LC*_50_ value of Bio-AgNPs was determined to be 56.5 μg/ml, indicating that they are highly toxic to *A. salina*. Kummara et al. (2016) reported that Bio-AgNPs were less toxic to human red blood cells and brine shrimp than AgNPs synthesized chemically. In conclusion, the findings of this study open up a new avenue for the use of marine-derived AgNPs, which have significant antimicrobial, antioxidant, insecticidal, and anticancer potential.

## Data Availability Statement

The datasets presented in this study can be found in online repositories. The names of the repository/repositories and accession number(s) can be found in the article/Supplementary Material (https://www.ncbi.nlm.nih.gov/nuccore/KY772427.1?report=GenBank, accession number: KY772427).

## Author Contributions

MK: conceptualization, methodology, formal analysis, data curation, and writing–review and editing. AE-S: conceptualization, validation, and visualization. MA: methodology and investigation. FA: methodology and preparation. SM: investigation and formal analysis. JS: investigation, visualization, and validation. SA: formal analysis, data curation, and writing–review and editing. All authors contributed to the article and approved the submitted version.

## Conflict of Interest

The authors declare that the research was conducted in the absence of any commercial or financial relationships that could be construed as a potential conflict of interest.

## Publisher’s Note

All claims expressed in this article are solely those of the authors and do not necessarily represent those of their affiliated organizations, or those of the publisher, the editors and the reviewers. Any product that may be evaluated in this article, or claim that may be made by its manufacturer, is not guaranteed or endorsed by the publisher.

## Footnotes

1
http://www.ncbi.nlm.nih.gov/BLAST/

2
https://www.ncbi.nlm.nih.gov/nuccore/KY772427.1?report=GenBank

## Supplementary Material

Supplementary Figure 1 Agar well diffusion technique on MHA plates showing antibacterial activity of Bio-AgNPs against selected isolates **(A)** *S. aureus* and **(B)** ESβL *E. coli*. **(1)** Bio-AgNPs (50 μg/ml). **(2)** Tested antibiotic alone (piperacillin-tazobactam, TZP). **(3)** TZP and Bio-AgNPs (50 μg/ml) in combination. **(4)** Bio-AgNPs (200 μg/ml). **(5)** TZP and Bio-AgNPs (200 μg/ml) in combination.
