# Supplementary figures and images for: Biosynthesis of Silver Nanoparticles by Marine Actinobacterium Nocardiopsis dassonvillei and Exploring Their Therapeutic Potentials
Source: Front Microbiol. 2022 Feb 3;12:705673. doi: 10.3389/fmicb.2021.705673 (PMC8862148; doi:10.3389/fmicb.2021.705673)

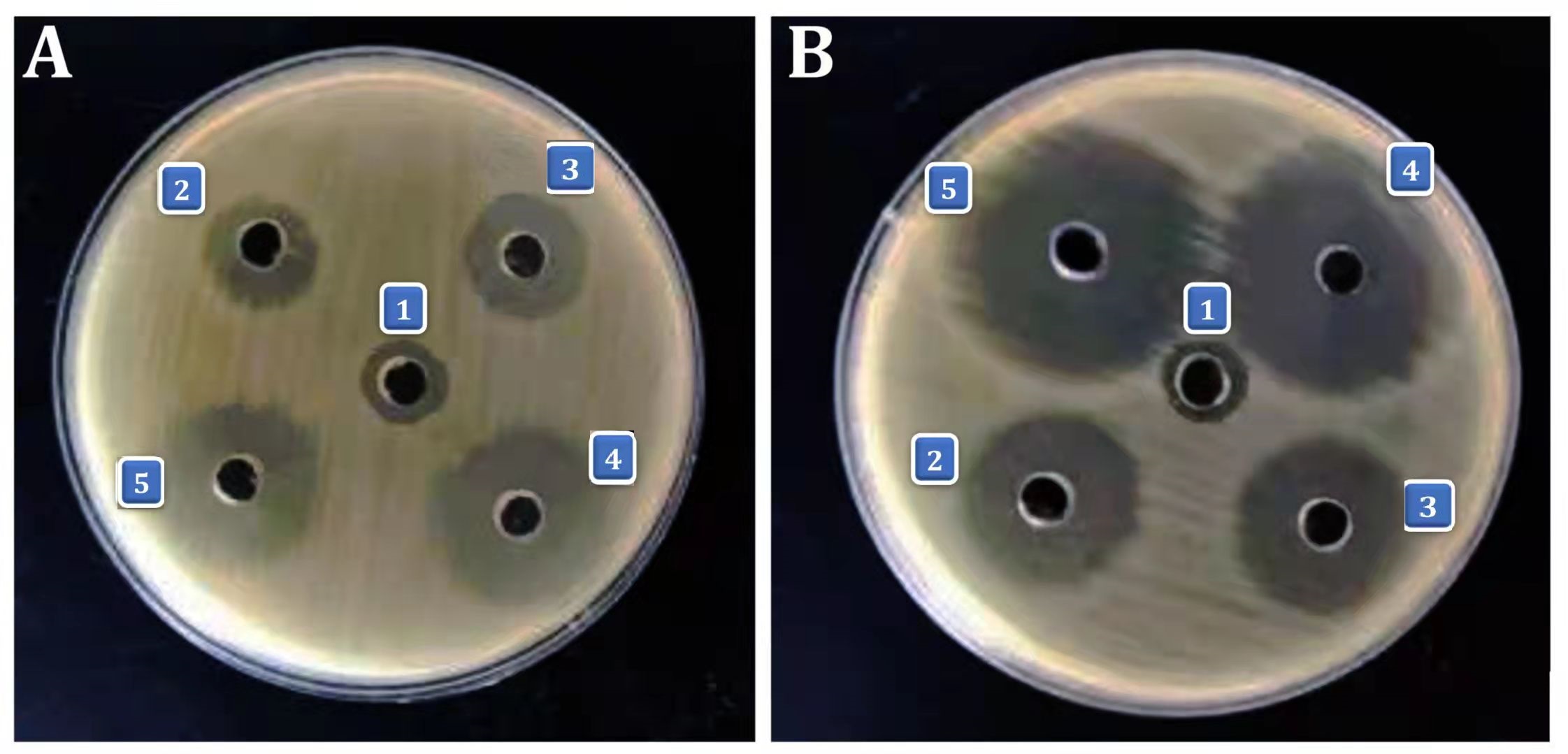

Supplement: Supplementary Figure 1 — Agar well diffusion technique on MHA plates showing antibacterial activity of Bio-AgNPs against selected isolates (A) S. aureus and (B) ESβL E. coli. (1) Bio-AgNPs (50 μg/ml). (2) Tested antibiotic alone (piperacillin-tazobactam, TZP). (3) TZP and Bio-AgNPs (50 μg/ml) in combination. (4) Bio-AgNPs (200 μg/ml). (5) TZP and Bio-AgNPs (200 μg/ml) in combination. [file Image_1.jpg]
